# Supplementary material for: Seminoma and Embryonal Carcinoma Footprints Identified by Analysis of Integrated Genome-Wide Epigenetic and Expression Profiles of Germ Cell Cancer Cell Lines
Source: PLoS One. 2014 Jun 2;9(6):e98330. doi: 10.1371/journal.pone.0098330 (PMC4041891; doi:10.1371/journal.pone.0098330)
Supplement: File S1 — ZIP file containing DMRforPairs output for significant regions. Please start from the html files. (ZIP) [file pone.0098330.s008.zip › figures/330.pdf]

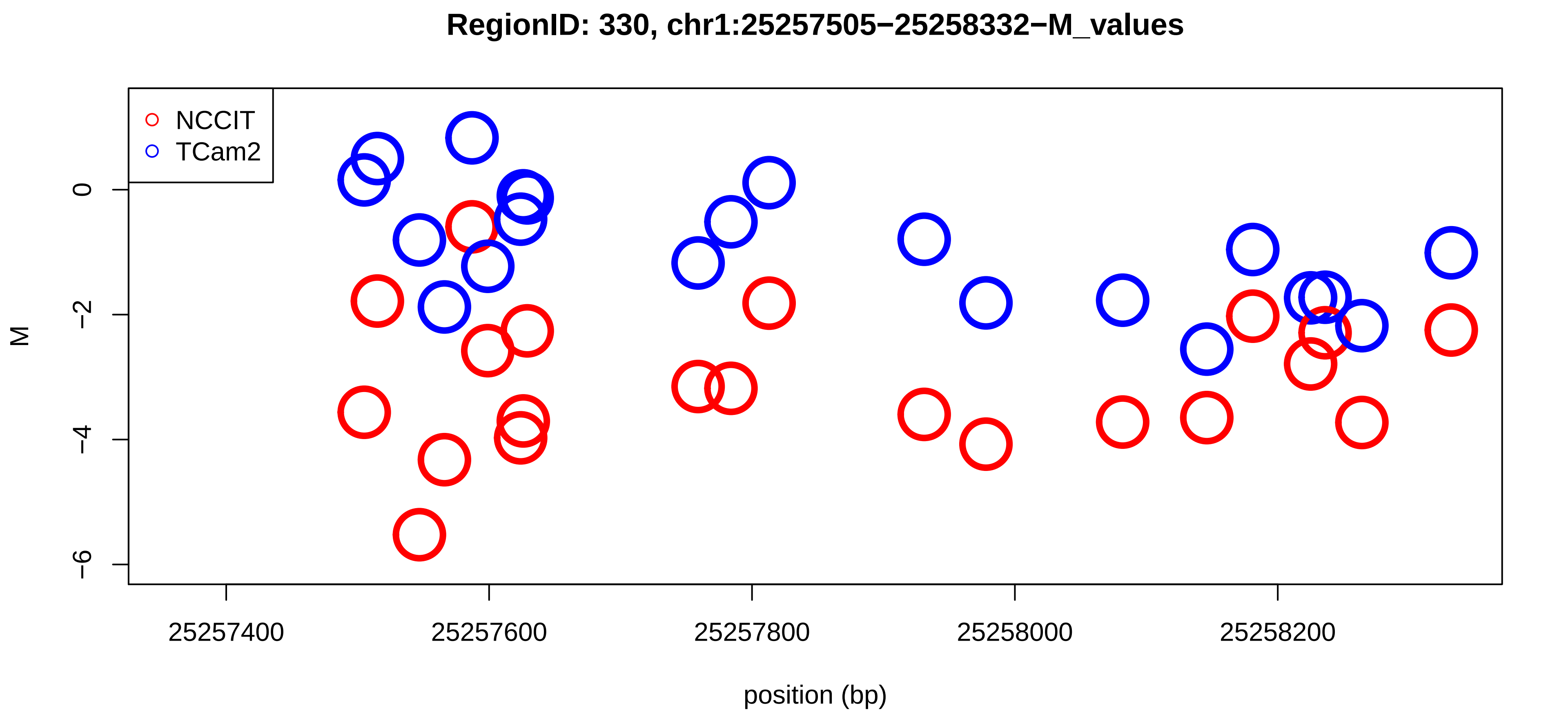
RegionID: 330, chr1:25257505−25258332−M_values
NCCIT
TCam2
M
0
−2
−4
−6
25257400
25257600
25257800
25258000
25258200
position (bp)

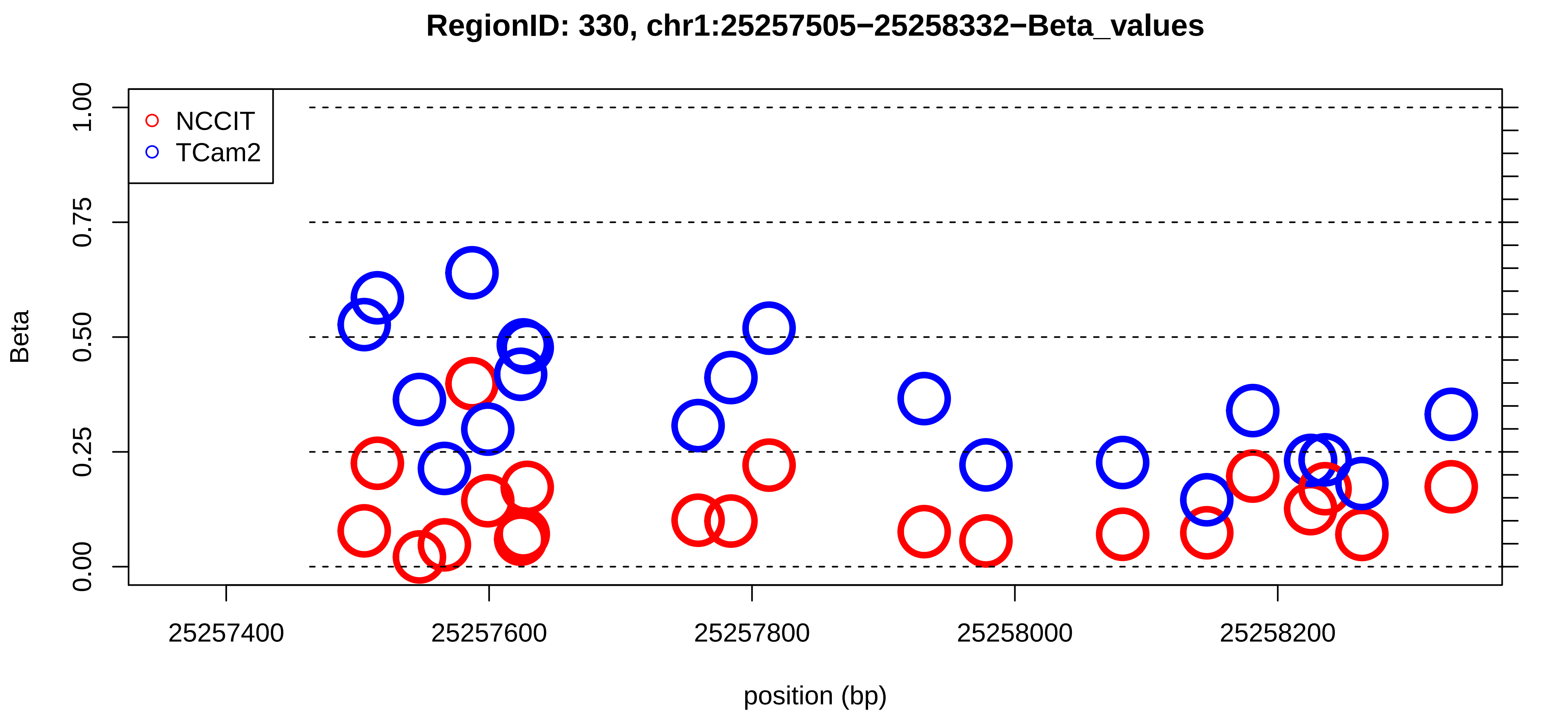
RegionID: 330, chr1:25257505−25258332−Beta_values
NCCIT
TCam2
Beta
0.00
0.25
0.50
0.75
1.00
25257400
25257600
25257800
25258000
25258200
position (bp)

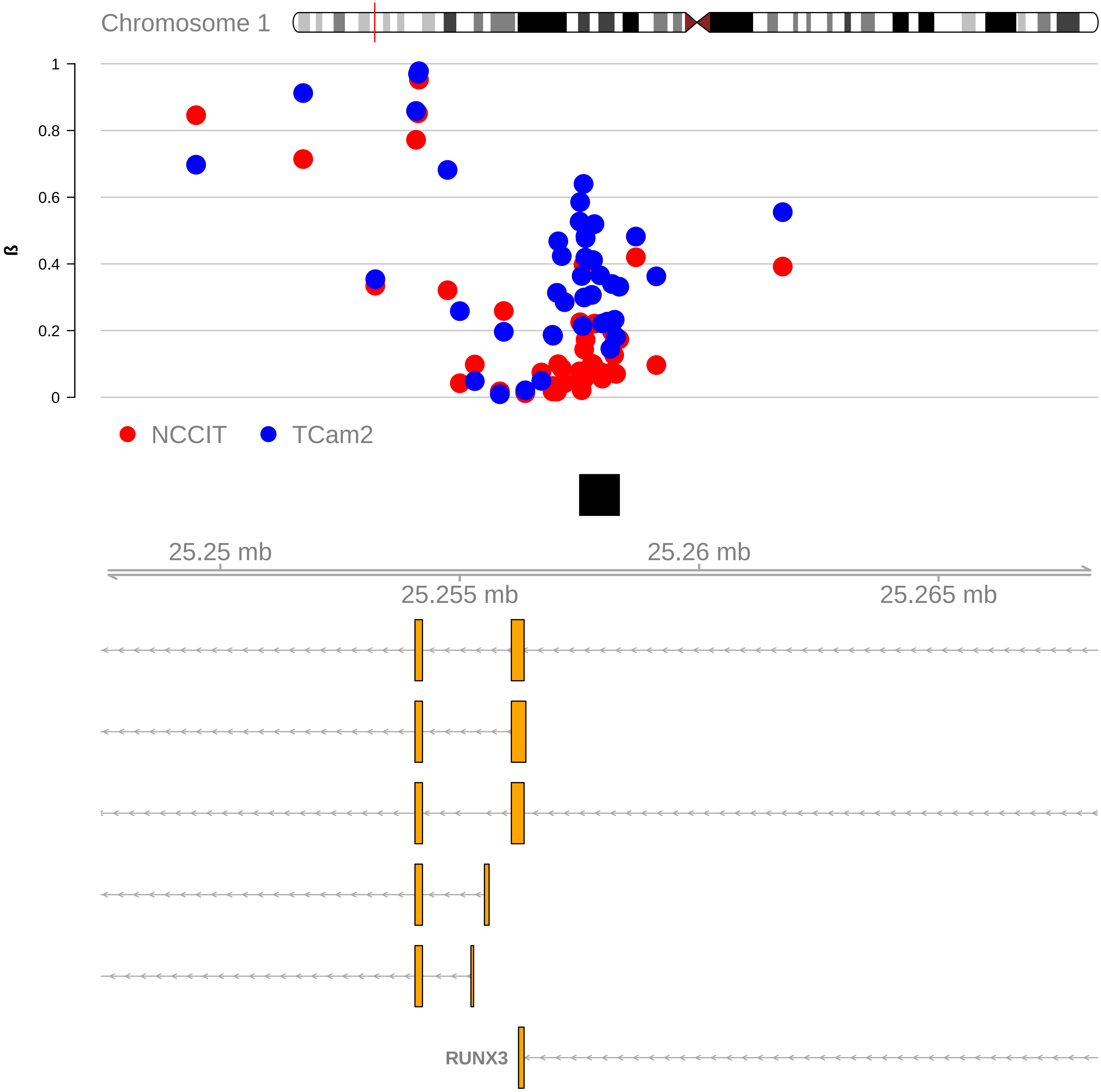
Chromosome 1
β
1
0.8
0.6
0.4
0.2
0
NCCIT
TCam2
25.25 mb
25.26 mb
25.255 mb
25.265 mb
RUNX3
